# Influence of Social Determinants of Health on COVID-19 Infection in Socially Vulnerable Groups

**DOI:** 10.3390/ijerph19031294

**Published:** 2022-01-24

**Authors:** Wladimir Morante-García, Rosa María Zapata-Boluda, Jessica García-González, Pedro Campuzano-Cuadrado, Cristobal Calvillo, Raquel Alarcón-Rodríguez

**Affiliations:** 1 Doctors of the World, 04001 Almería, Spain; wladimir.morante@medicosdelmundo.org; 2 Department of Nursing, Physiotherapy and Medicine, University of Almeria, 04120 Almería, Spain; rzapata@ual.es (R.M.Z.-B.); pozy.calvillo@gmail.com (C.C.); ralarcon@ual.es (R.A.-R.); 3 Doctors of the World, 28028 Madrid, Spain; pedro.campuzano@medicosdelmundo.org

**Keywords:** coronavirus, epidemiology, healthcare disparities, poverty areas, public health, socioeconomic factors

## Abstract

The coronavirus 2019 (COVID-19) pandemic has had a significant impact on the economy and health, especially for the most vulnerable social groups. The social determinants of health are one of the most relevant risks for becoming infected with COVID-19, due to the health consequences for those who are exposed to it. The objective of this study was to analyze the influence of social determinants in health on COVID-19 infection in vulnerable social groups. A transversal epidemiological study was carried out on 746 individuals in vulnerable situations living in conditions of extreme poverty in disadvantaged areas in the province of Almeria (southeast of Spain). Social determinants of health such access to drinking water (*p* < 0.001) and economic income (*p =* 0.04) influenced the infection of COVID-19. A binary logistic regression model showed that the significant predictors of COVID-19 infection were the lack of economic income and inaccessible drinking water. The government and social health services must be aware of this problem in order to play an active role in searching for solutions and implementing public health prevention measures to eliminate social inequalities in health.

## 1. Introduction

The illness caused by coronavirus 2019 (COVID-19) continues to advance at a global level [1], affecting a large number of people [2]. According to the World Health Organization (WHO), up to 27 December 2021, 279,114,972 COVID-19 cases have been confirmed, including 5,397,580 deaths worldwide. In Spain, there are 5,718,008 cases confirmed and 89,019 deaths of COVID-19 [3], observing significant variations in the evolution of the pandemic. From an administrative point on view, Spain is organized in 17 autonomic regions, with an independent health administration in each one. Almería, that belongs to Andalusia autonomic region, is located in the southeast of Spain and has 201,780 inhabitants. COVID-19 pandemic has had a big impact in its population, with 78,487 confirmed cases up to the moment this report is being written [4].

As described in previous public health crises, pandemics do not affect people uniformly [5]. The COVID-19 pandemic has had a significant impact on the economy and health, especially for the most vulnerable social groups [6]. As the pandemic continues, reports are emerging of clinical outcomes and risk factors for admission and mortality in the intensive care unit [7].

Among the risk factors associated with the COVID-19 disease and its evolution are cardiovascular and respiratory disease, high blood pressure, and diabetes [8]. In addition, other conditions such as oncological diseases and chronic liver disease have been reported frequently in hospitalised infected patients [9]. The potential relationship between death and various ethnic characteristics and social determinants of health (unfair and avoidable health differences between population groups defined socially, economically, demographically, or geographically) [10] has also been considered, such as socioeconomic level, sociocultural factors, and type of housing [11].

Recent reports have described an increase in mortality related to COVID-19 in migrants and ethnic minorities in various western countries [12,13,14,15,16,17]. However, the clinical outcomes on mortality associated with ethnic minorities are related to their socioeconomic situation, comorbidity, and the unequal access to medical services [18,19,20,21]. A study carried out in Spain highlights that the lower the average economic income, the higher incidence of COVID-19. Furthermore, it specifies that the district with the lowest incomes had an incident rate of the disease 2.5 times higher than the district with the highest incomes [22]. Those who live in marginalised neighbourhoods or shanty towns have been studied in different low- and middle-income countries, as contexts in which there have been a greater number of COVID-19 infections, being places where there are vulnerabilities in various social determinants of health such as housing, precarious work, and lack of running water [23,24,25]. Additionally, space constraints, violence, and overcrowding in marginalised neighbourhoods make social distancing and self-quarantine impractical. As a result, the rapid spread of an infection is highly likely [23,26,27].

In this context, inequalities in the risk of COVID-19 infection are to be expected, with the most vulnerable groups having a higher risk of infection. Contextualizing this problem in Spain, it is important to highlight that Almería informal settlements inhabitants are mostly of Moroccan, sub-Saharan and Spanish nationality. According to Doctors of the World Andalusia’s report, more than 3500 people live in about 120 informal settlements in Almeria [28], especially in the area of “El Ejido”, “Nijar” and “Roquetas de Mar” [29], which represents an increase of about 20% over the last few years. Due to the immigrant inflow, mostly from the African continent, an important number of immigrants arrived in Spain in small boats and hidden in trucks looking for greenhouse jobs. Almería province is known by its intensive agriculture and by providing vegetables to most of Europe. This makes people want to come to Almería looking for a better future. These immigrants end up living in farmhouses close to the greenhouses, shared apartments, abandoned warehouses and shantytowns, where they live crammed. Power is illegally taken from the distribution network and water is taken from pools (stored in recycled cans taken from nearby greenhouses). On several occasions, they suffer the stigma from natives, the shantytowns are away from towns, which makes accessing health and social services difficult for them. Almería’s underprivilege areas are characterized by [28]:

1. Damaged houses and lack of infrastructure, equipment and public services.
2. High rates of absenteeism and dropout.
3. High rates of unemployment and lack of training.
4. Significant hygienic-sanitary deficiencies.

Furthermore currently, few studies exist on the potential role of social determinants of health in the risk of COVID-19 infection in vulnerable populations, particularly in Spain. For this reason, the objective of this study was to analyze the influence of social determinants of health on the COVID-19 infection in vulnerable social group in the province of Almería (Spain).

## 2. Materials and Methods

### 2.1. Study Design

A cross-sectional epidemiological study was carried out on people in vulnerable situations living in marginalised neighbourhoods or shanty towns in the province of Almeria (southeast of Spain) in order to analyse the influence of social determinants of health on the spread of COVID-19.

### 2.2. Study Population and Data Collection

The study population consisted of 746 individuals from vulnerable groups living in three unfavorable areas in the province of Almería: “Níjar”, “El Ejido” and “Roquetas de Mar”.

The data were collected during the period of time from June to December 2020. The participants in this study were recruited through the “Doctors of the World” association in Spain. “Doctors of the World” is an independent association that works to make the right to health effective for all people, especially for vulnerable or excluded populations, and victims of natural disasters, famines, diseases, armed conflicts, or political violence. The association is formed by volunteers and workers who carry out interventions aimed at healthcare for vulnerable or excluded populations or victims of crisis, whose right to access health has been violated.

Data was recollected by a paper survey performed by “Doctors of the World” nurses. This association regularly attends (twice a week) the disadvantaged areas throughout the whole year to offer health assistance to vulnerable people. During the visits to the three informal settlements in Almería, nurses recollected the data and performed the quick tests. The average time of completion of the final questionnaire was thirty minutes approximately. Furthermore, those nurses have a basic knowledge of different languages, like Arabic, Spanish, English and French, something that facilitated the task of data recollection and the understanding with the study participants. Finally, the survey was applied depending on the participant’s language. The nurses used a paper version.

During the pandemic, “Doctors of the World” not only carried out interventions at the biopsychosocial level for this type of population (referrals to hospital centres for health problems, donation of food and drinking water, health training, and psychological interventions), but also performed COVID-19 infection tests. The contagion tests were rapid tests to determine if antibodies were present in the blood (antibodies against IgM and IgG). Positive results from the antibody test were confirmed by a PCR test.

The inclusion criteria were people over 18 years old, residents of marginalised neighbourhoods of Almería and those that voluntarily agreed to participate in the study. The exclusion criteria included minors and those who declined to participate in the study.

The variables included in the study were the following sociodemographic variables: age, sex, nationality, primary studies, consumption of substances (tobacco, cannabis, or alcohol), having a partner, having children, a regular administrative situation, variables related to social determinates of health: type of housing, access to drinking water, access to electricity, overcrowding, economic income, difficulty to access the Public Health System, and rapid blood antibody determination tests for COVID-19.

### 2.3. Data Analysis

A database was created to store the collected data. The data was analysed through the statistical software IBM SPSS version 26.0 (IBM Inc., Armonk, NY, USA). A descriptive analysis was carried out and the means and standard deviations (SD) were calculated for the continuous variables, and the absolute and relative frequency distributions were calculated for the categorical variables. For the comparison of qualitative variables, the chi-square test (χ^2^) was used. The ORs with their corresponding 95% CI were calculated. To compare the means of the quantitative variables, after performing a normality test (Kolmogorov-Smirnov test), non-parametric tests were applied; the Mann–Whitney U test to compare the independent variables. A multivariate analysis was performed using binary logistic regression. The level of statistical significance was set at a value of *p* < 0.05.

### 2.4. Ethical Considerations

Approval for this study was obtained from the Ethics and Research Commission of the University of Almería (ID: EFM 145/2021). All the procedures were performed in accordance with the ethical standards of the Helsinki Declaration. Participation in the study was voluntary. All participants signed the informed consent form.

## 3. Results

A total of 746 individuals participated who were in vulnerable social situations and lived in conditions of extreme poverty in marginalised neighbourhoods. The participants were divided into two groups; those who had a positive result from the COVID-19 antibody test COVID-19 (*n* = 84) and those who had a negative result from the COVID-19 antibody test (*n* = 662).

The study groups did not differ in regard to possible confounding factors such as age, gender, primary education, consumption of substances (tobacco, cannabinoids and alcohol), having a partner, having children, and a regular administrative situation (Table 1).

Regarding nationality, the positive cases of COVID-19 occurred in 60.7% (*n* = 51) of the Moroccan population, in 26.2% (*n* = 22) of the sub-Saharan population and in 13.1% (*n* = 11) of the Spanish population, these differences being statistically significant.

Upon analysing the social determinants of health, the majority of the COVID-19 cases occurred in those who lived in homes occupied by squatters (51.2%), in overcrowded conditions (57.1%), with access to electricity (91.7%), and 14.3% had difficulty accessing the Public Health System (Table 2). No statistically significant differences were observed when comparing these social determinants to those who tested positive or negative for COVID-19.

Of the positive COVID-19 cases, 51% did not have any type of income and only 3.6% of these cases had access to drinking water. These results were statistically significant when compared to both those who tested positive and negative for COVID-19.

When considering the dependent variable as having or not having tested positive for COVID-19, and the independent variables being age, gender, access to safe drinking water and economic income, the results obtained from the multiple logistic regression analysis showed the influence of these social determinants of health on COVID-19 infection.

Those who had no economic income showed a significantly greater risk of testing positive for COVID-19 (OR: 2.26). The group who had no access to safe drinking water also had a greater risk of COVID-19 infection, observing an OR of 9.23 (Table 3).

## 4. Discussion

The influence of social determinants of health on COVID-19 infection has generated enormous concern in Spain [30]. This study intends to analyse the influence of social determinants of health on the spread of COVID-19 in vulnerable social groups.

### 4.1. Influence of Sociodemographic Variables

According to the model of social inequalities in health [31] some factors such as sex [32], ethnicity, social class, or place of residence [10,11,17,33] act as axes of inequality, causing differentiated access to resources and generating inequity in health outcomes.

In the context of Spain, one study [33] found a higher risk of contracting the COVID-19 disease in people from sub-Saharan Africa, the Caribbean, and Latin America compared to those from Spain, migrants from Europe or from the north of Africa. Another study carried out in Spain shows a greater impact of COVID-19 on migrants from Latin America [17]. Despite these results, it should be noted that ethnic origin is a complex concept that is composed of genetics, social constructions, cultural identity, and behavioural patterns [34].

In this study, age, having a partner, having children, an irregular administrative situation, and level of studies did not show significant differences in relation to a higher prevalence of COVID-19 infections. Other studies have shown a greater exposure to COVID-19 infection in irregular migrants [35]. However, it is possible that these differences with respect to the administrative situation do not carry much weight in a more homogeneous group of people who live in shanty towns and work in similar precarious conditions.

### 4.2. Influence of the Social Determinants of Health

Inequalities in housing conditions may also contribute to inequalities in COVID-19 infection by increasing COVID-19 transmission rates [36]. Previous studies indicate that the quality of housing, including the level of occupancy, access to electricity, drinking water, etc., are variables closely related to a higher risk of COVID-19 infection [10,36,37,38,39]. In our study, no statistically significant differences were observed in regard to housing type. This may be due to the fact that our study focuses only on groups in vulnerable situations who live in informal settlements. These people live in different types of housing (shacks, squatter houses, and houses) in a precarious situation. Those who live in a “house” are also considered to be living in a precarious situation, similar to a “shack” and “squatter house”, because they are houses without the basic needs of water, bathrooms, sewage, drainage, garbage collection, etc. In most cases there are space limitations and multiple occupancy is common. For this reason, it is possible that the housing type in our study is not significant in relation to the COVID-19 test result, since in the three housing types (shack, squatter house and house) we observed similar situations.

Furthermore, the lack of running water in homes is an important factor when determining the risk of COVID-19 infection [37]. In our case, statistically significant differences were found, which confirm the strong association (OR: 9.23; IC: 2.81–30.28) between inaccessible drinking water and the risk of COVID-19 infection. The low socioeconomic level could lead to inadequate living conditions such as lack of electricity or water [36]. In order to develop a public health investigation, it is necessary to implement policies and programmes that reduce housing inequalities (including access to drinking water, electricity, etc.) which affect the wellbeing of marginalised neighbourhoods or shanty towns where vulnerable social groups live [37]. The people who live in informal settlements in Almeria get their water for daily consumption from outdoor fountains and irrigation pools. This water is normally used to irrigate crops in nearby greenhouses (intensive farming areas). During the pandemic and in the periods of confinement, these people have had more difficulty moving around and accessing water. This situation has meant that the preventative hygiene measures recommended by the WHO (such as continuous hand washing, cleaning daily, etc.) were not maintained, which could have influenced COVID-19 infection [37].

The binary logistic regression model shows that a relevant association exists (OR: 2.26; CI: 1.41–3.62) between not having economic resources with a greater risk of becoming infected with COVID-19. These results coincide with those found in other studies that indicate that individuals with low socioeconomic status have a greater exposure to poor quality, unsafe, or unaffordable housing and therefore, have a higher rate of negative health consequences, including COVID-19 infection [10,22,40,41]. Similar results to ours were described in another study carried out in Spain, indicating that there is a greater prevalence of COVID-19 infection in low-paid employees living in disadvantaged areas. Furthermore, they stated that workers with low wages, the unemployed, and individuals who no longer receive unemployment benefits, had a higher probability of becoming infected with COVID-19 than workers with wages of † 18,000 per year [42]. Moreover, the lack of economic resources is another indicator of other social determinants of health in vulnerable populations, due to having to accept more precarious or insecure jobs and worse living conditions [36]. Marmot et al. [43] highlighted that the COVID-19 pandemic exposes and amplifies existing inequalities in society. This requires the implementation of coordinated measures of control, diagnosis, and treatment of the pandemic, with the aim to avoid an increase in inequality, as well as the identification of vulnerable groups that require more economic assistance to recover from the pandemic [42]. It should be noted that disease control efforts should be more intense in areas where the most vulnerable population lives [22], guaranteeing adequate accessibility to diagnosis and treatment. We find disparities in the COVID-19 infection risk, with the most vulnerable group having a higher risk. For this reason, it is important to understand the impact that social inequalities have on COVID-19 infection risk, to develop strategies aimed at reducing COVID-19 incidence, with the goal of mitigating pandemic social effects.

Based on our results, it is necessary to show the effects of economic income and access to drinking water on social vulnerability, around three conceptual models. (1) Birkmann [44] designed a conceptual model called the Key Spheres of the Concept of Vulnerability. This model is based on five hierarchical levels and each level corresponds to a different concept of vulnerability [44,45]. In the explanation of the five levels of the model, Birkmann [44] argues that the fifth level suggests that vulnerability not only refers to aspects of the person, but also the issues and parameters that determine vulnerability, such as economic, social, contextual or institutional characteristics. This indicates that emphasis is placed on an interdisciplinary analysis of the multidimensional concept of vulnerability. (2) Flaskerud and Winslow [46] developed the Vulnerable Populations Conceptual Model. This model proposes that (a) the availability of resources, (b) the relative risk, and (c) the state of health (morbidity and mortality), are related [46]. It is worth mentioning that, for this model, the availability of resources refers to the availability of socioeconomic and environmental resources, such as salary, work, education, social status, housing, access to water, family support, access to quality healthcare, etc. [46,47]. (3) On the other hand, there is a more recent model proposed by Shi et al. [48] called the General Model of Vulnerability. This model highlights the importance of determinants of vulnerability at the contextual level, whereby vulnerability is not only determined by personal characteristics, but it is also determined by the context, over which individuals have little or no control, and by the interaction between the individual and the context [48,49]. This avoids blaming the victim and highlights the importance of intervention at the social level [48]. Another important point about this model is that risk factors, both individual and contextual, are determined by a convergence of predisposing, enabling, and necessity characteristics. Shi et al. [48] mentions that predisposing characteristics are those that are based on the propensity of individuals to use healthcare services, considering basic demographic variables such as age and/or sex, social variables such as ethnicity, education and/or employment, and aspects related to beliefs about health. Enabling characteristics refer to the means available to individuals and the attributes of the community in which a person lives to access health services, such as income, insurance coverage, and/or the availability of healthcare services. Finally, the characteristics of need are those specific diseases or needs in relation to health, which are the main causes that motivate healthcare assistance [48,49]. Although these three models mentioned above explain vulnerability differently, it is important to stress that this concept constitutes a human condition in which the individual does not have the appropriate skills to deal with a great threat or harm. Therefore, it is essential to identify and consider the personal and contextual characteristics that put the individual at risk in order to improve their physical, mental, and social well-being.

### 4.3. Strengths and Limitations

The main strength of this study was that it enabled an analysis to be carried out that relates the social determinants of health and their possible influence on the increased risk of infection and spread of COVID-19 in vulnerable populations. To date, few studies have been carried out with a similar analysis in vulnerable social groups in Spain. Another strength of this study was the relatively high number of participants included in a context that is difficult to access. On the other hand, the main limitations include the difficulty of accessing vulnerable populations in marginalised neighbourhoods and the language barrier. In addition, another limitation of this study was the lower participation of the female population, which may be due to the cultural beliefs of a high percentage of the individuals who reside in marginalised neighbourhoods in south-eastern Spain.

## 5. Conclusions

The results of this study indicate that social determinants of health influence the increased risk of infection and development of COVID-19 in vulnerable populations. The social determinants of health that carry the most risk include access to safe drinking water and economic income. The binary logistic regression model showed that the significant predictors of COVID-19 infection were having no economic income, and inaccessible safe drinking water. For this reason, the government and social and health services must be aware of this problem and take on an active role in searching for solutions and implementing public health prevention measures to eliminate social inequalities in health.

## Figures and Tables

**Table 1:** Analysis of sociodemographic variables among COVID-19 positive and negative individuals.

Variables		COVID-19 Test Result		
		Positive (*n* = 84)	Negative (*n* = 662)	
		M (SD)	M (SD)	*p*-Value
Age (years)		40.33 (13.81)	40.08 (15.20)	0.88 ^a^
		n (%)	n (%)	
Gender	Male	57 (67.9)	390 (58.9)	0.11 ^b^
	Female	27 (32.1)	272 (41.1)	
Nationality	Moroccan	51 (60.7)	364 (56.5)	<0.001 ^b^
	Spanish	11 (13.1)	197 (30.6)	
	Sub-Saharan	22 (26.2)	9 (12.9)	
Primary studies	Yes	46 (54.8)	379 (57.3)	0.66 ^b^
	No	38 (45.2)	283 (42.7)	
Tobacco	Yes	10 (11.9)	114 (17.2)	0.21 ^b^
	No	74 (88.1)	548 (82.8)	
Cannabinoids	Yes	2 (2.4)	33 (5)	0.28 ^b^
	No	82 (97.6)	629 (95)	
Alcohol	Yes	10 (11.9)	80 (12.1)	0.96 ^b^
	No	74 (88.1)	582 (87.9)	
Partner	Yes	51 (60.7)	364 (55)	0.31 ^b^
	No	33 (39.3)	298 (45)	
Children	Yes	60 (71.4)	412 (62.2)	0.10 ^b^
	No	24 (28.6)	250 (37.8)	
Regular administrative status	Yes	62 (73.8)	486 (73.4)	0.93 ^b^
	No	22 (26.2)	176 (26.6)	

*p* value obtained using ^a^ Mann-Whitney U test for continuous variables or ^b^ Chi-squared test for categorical variables.

**Table 2:** Analysis of the social determinants of health among COVID-19 positive and negative individuals.

Variables		COVID-19 Test Result		
		Positive (*n* = 84)	Negative (*n* = 662)	
		M (SD)	M (SD)	*p*-Value
		n (%)	n (%)	
Type of housing	Shack	3 (3.6)	89 (13.4)	0.95
	Squatted house	43 (51.2)	304 (45.9)	
	House	38 (45.2)	269 (40.6)	
Access to safe drinking water	Yes	3 (3.6)	126 (19)	<0.001
	No	81 (96.4)	536 (81)	
Access to electricity	Yes	77 (91.7)	590 (89.1)	0.47
	No	7 (8.3)	72 (10.9)	
Overcrowding	Yes	48 (57.1)	334 (50.5)	0.24
	No	36 (42.9)	328 (49.5)	
Economic income	Low	41 (49)	406 (61.2)	0.04
	None	43 (51)	256 (38.8)	
Difficulty of access to the Public Health System	Yes	12 (14.3)	119 (18)	0.40
	No	72 (85.7)	543 (82)	

*p*-value obtained using Chi-squared test for categorical variables.

**Table 3:** Stepwise multiple logistic regression analysis of positive COVID-19 adjusted for social determinants of health.

Parameters	OR	95% C.I.	*p*-Value
No economic income	2.26	1.41–3.62	0.001
No access to safe drinking water	9.23	2.81–30.28	0.001

The following variables were entered into the model: age, gender (0: female, 1: male), access to safe drinking water (0: yes, 1: no) and economic income (0: low, 1: none). Goodness-of-fit: Nagelkerke R-Square was 0.68 and *p*-value for Hosmer-Lemeshow test was 0.25.

## Data Availability

Data of this study are stored in an SPSS software Project.
